# Elevated insulin-like growth factor 2 mRNA binding protein 1 levels predict a poor prognosis in patients with breast carcinoma using an integrated multi-omics data analysis

**DOI:** 10.3389/fgene.2022.994003

**Published:** 2022-08-24

**Authors:** Shiqi Li, Meixiu Jiang

**Affiliations:** ^1^ Queen Mary School, Nanchang University, Nanchang, China; ^2^ The National Engineering Research Center for Bioengineering Drugs and the Technologies, Institute of Translational Medicine, Nanchang University, Nanchang, China

**Keywords:** IGF2BP1, BRCA, prognosis, enrichment analysis, differentially expressed genes, genetic correlation

## Abstract

**Background:** Insulin-like growth factor 2 mRNA binding protein 1 (IGF2BP1) controls the cytoplasmic fate of certain mRNAs and is hypothesized to predict a poor patient prognosis in several malignant tumors. However, the prognostic relevance of IGF2BP1 in breast cancer remains debatable.

**Methods:** We interrogated large publicly available datasets from the Gene Expression Omnibus (GEO), The Cancer Genome Atlas (TCGA), and cBioportal databases to analyze the genetic alterations in the expression levels of IGF2BP1 in patients with invasive breast carcinoma (BRCA), and to discern the prognostic value of IGF2BP1 in BRCA. We applied Gene Ontology (GO), the Kyoto Encyclopedia of Genes and Genome (KEGG), and gene set enrichment analysis (GSEA) to uncover a functional association between IGF2BP1 and BRCA using differentially expressed genes (DEGs), and we screened genes and proteins related to BRCA.

**Results:** We determined that both genetic alterations in IGF2BP1 (approximately 10%) and an increase in IGF2BP1 mRNA levels were related to certain cancer subtypes and an unfavorable prognosis in BRCA patients, and we then established an OS nomogram upon our multivariate regression model. The DEGs and IGF2BP1-correlated genes/proteins that implied the involvement of cornification, keratinization, drug/xenobiotic metabolism by cytochrome P450, chemical carcinogenesis, cell interactions, and cell adhesion to the extracellular matrix (ECM) pathways with respect to the prognostic relevance of IGF2BP1.

**Conclusion:** In summary, our results indicated that both genetic alterations in IGF2BP1 and increased levels of IGF2BP1 mRNA and protein predict a poor patient prognosis in BRCA patients.

## 1 Introduction

Breast cancer constitutes the most prevalent form of cancer found in women globally, and its incidence in China is rising annually in recent years. It is firmly established that breast cancer is a biologically heterogeneous disease, and four intrinsic molecular subtypes (luminal A, luminal B, HER2-positive, and basal-like) facilitate prognostication. However, although prediction of therapeutic has improved survival over the past 30 years relative to clinicopathologic categories, breast cancer remains one of the leading causes of cancer death in women principally due to recurrence, metastasis, or therapeutic failure (e.g., with acquired drug resistance to tamoxifen). Given the heterogeneous clinical outcomes of any one subtype, the challenge is now to identify tumors that portend a less favorable prognosis. Growth, metastasis, and even responses to breast cancer therapy are closely related to deregulation, mutation, and epigenetic mechanisms with respect to certain genes, and the use of integrated multi-omics such as integrated genomics, transcriptomics, and proteomics is thus critical to providing prognostic factors and to refining clinically relevant subtypes by data-mining (Perou et al., 2000; Sorlie et al., 2003).

IGF2BP1—also known as IMP1, ZBP1, CRDBP, and VICKZ1—belongs to the insulin-like growth factor 2 mRNA-binding protein family, the IGF2BPs. The proteins in this family bind to the mRNAs of certain genes and regulate their translation, decay, and transport, or promote the formation of “stable” protein–mRNA complexes (Huang et al., 2018a). There are still numerous unidentified target mRNAs for IGF2BP1—including ACTB, CTNNB1, GLI1, IGF2, MAPK4, MDR1, PPP1R9B, CD44, MYC, PTEN, and BTRC which indicate that this protein modulates multiple important aspects of cellular function during both normal development and cancer (Bell et al., 2013). In fact, IGF2BP1 has been found to be frequently overexpressed in many cancers, such as head and neck squamous cell carcinoma (HNSCC) (Paramasivam et al., 2021), melanoma (Kim et al., 2018; Mahapatra et al., 2019), cervical cancer (Wang et al., 2018), ovarian carcinoma (Kobel et al., 2007; Mahapatra et al., 2017) and lung cancer (Shi et al., 2017). Because of its strong correlation with unfavorable prognosis and drug resistance, IGF2BP1 is also considered one of the most promising therapeutic targets (Bell, et al., 2013; Huang et al., 2018b; Kim, et al., 2018).

A role for IGF2BP1 in breast cancer, however, remains controversial. Some investigative groups reported that IGF2BP1 was downregulated and that it suppressed the invasive phenotype of human breast carcinoma cells both *in vitro* (Gu et al., 2012) and in a mouse xenograft model through the regulation of its target mRNAs (Wang et al., 2016) or *via* an interaction with the lncRNA urethral carcinoma-associated 1 (UCA1) (Zhou et al., 2018). In contrast, another group reported that IGF2BP1 was reactivated in breast cancer cells by beta-catenin, and that this interaction stabilized beta-catenin (Gu et al., 2008). To clarify this area of focus, we analyzed the functional relationships between IGF2BP1 and clinical values in patients with invasive breast carcinoma (BRCA) *via* data mining of multiple databases and enrichment analysis.

## 2 Materials and methods

### 2.1 Correlation between genetic alterations in insulin-like growth factor 2 mRNA binding protein 1 and breast cancer prognosis using cBioPortal

We analyzed the genomic profiles of IGF2BP1 using a dataset from the Molecular Taxonomy of Breast Cancer International Consortium (METABRIC) and retrieved an integrated genomic and transcriptomic targeted sequencing of 2,509 primary breast tumors and 548 matched normal tissues (Curtis et al., 2012; Pereira et al., 2016) in cBioPortal (www.cbioportal.org) (Gao et al., 2013). IGF2BP1 mRNA levels (RNA Seq V2 RSEM) were also determined and visualized by constructing a heatmap with cBioPortal. Comparisons of survival (overall survival [OS] or relapse free survival [RFS]) and clinical attributes between the genetically altered IGF2BP1 group and nonaltered group were then executed using genome data and clinical data in cBioportal.

### 2.2 Insulin-like growth factor 2 mRNA binding protein 1 expression levels in TCGA-breast carcinoma

RNAseq TPM data were obtained from UCSC XENA (https://xenabrowser.net/datapages/) for the TCGA and GTEx samples using the Toil pipeline (Vivian et al., 2017), and then log2-transformed. We evaluated the comparisons of IGF2BP1 expression in 33 types of human cancers and adjacent normal tissues (*n* = 15,776), in 1,109 unmatched BRCA tissues and 292 normal tissues, and in 112 paired BRCA tissues and adjacent normal breast issues. Gene-expression profiles of GSE7904 were further extracted from the GEO database to validate the expression of IGF2BP1 in BRCA.

### 2.3 Correlation analysis of insulin-like growth factor 2 mRNA binding protein 1 expression and clinical value

The histologic information and intrinsic molecular subtype information were retrieved from the TCGA database, and we conducted a correlation analysis of IGF2BP1 expression and breast cancer subtypes using ggplot2 (version 3.3.3) in R. We determined the differences between groups using the nonparametric Kruskal–Wallis test on ranks, and Student’s t-test or paired t-test for parametric data.

### 2.4 Correlation analysis of insulin-like growth factor 2 mRNA binding protein 1 expression with breast cancer prognosis

Our cohort including 1,082 patients was divided into low and high-expression subgroups according to the median level of IGF2BP1 mRNA. Kaplan–Meier curves for OS or/disease-specific survival (DSS) rates were generated using the survminer package (version 0.4.9) with gene expression data from TCGA-BRCA and the supplementary dataset from the TCGA pan-cancer clinical data resource (TCGA-CDR) (Liu et al., 2018). We utilized log-rank test to compare survival curves among high- and low- expression subgroups using the survival R package (version 3.2–10), and the differences of *p* < 0.05 were considered statically significant.

### 2.5 Creation of nomogram model

Univariate and multivariate Cox regression analyses were used to assess the effects of selected variables on OS. Variables that were significant upon univariate Cox regression analysis (*p* < 0.1) were subsequently subjected to multivariate Cox regression analysis, and we developed nomograms using the nomogram package in R with the independent predictors identified in the Cox proportional hazards model. The nomogram-predicted survival probabilities were compared with the observed survival probabilities by Cox analysis and visualized in the calibration curve using the rms (version 3.6.3) and survival packages (version 3.2–10) (Liu, et al., 2018).

### 2.6 Biological functional analysis and enrichment of differentially expressed genes

We downloaded and analyzed gene expression data of TCGA-BRCA in HTSeq-TPM, and compared the expression profiles (HTSeq-TPM) between the high- and low-IGF2BP1 mRNA expression groups so that to identify the DEGs with DESeq2 (version 1.26.0) (Love et al., 2014), applying filtering thresholds of |log2 fold-change| > 1.0 and adjusted *p* < 0.05. Then DEGs with |log2 fold change| > 2.0 and adjusted *p* < 0.05 were subjected to functional analysis with the clusterProfiler R package and the org. Hs.eg.db package for biological process GO terms and KEGG pathways (Yu et al., 2012). Gene set enrichment analysis (GSEA) against the MSigDB category (c2. cp.v7.2. symbols.gmt) was also performed to assess the enrichment of gene sets between IGF2BP1 high-expression and low-expression groups using the clusterProfiler R package (Version 3.14.3) (Subramanian et al., 2005). A false discovery rate (FDR) of <0.25 and p. adjust<0.05 were considered to reflect significant enrichment.

### 2.7 Screening of insulin-like growth factor 2 mRNA binding protein 1 correlated genes and proteins from different databases for breast carcinoma

Gene expression HTSeq-FPKM data from the TCGA-BRCA database were downloaded from TCGA, converted to TPM using the formula TPM = (FPKM ×106)/(sum of FPKM), and then log2 transformed. The IGF2BP1-correlated genes were screened with Pearson’s correlation coefficient (|r| >0.3 and *p* < 0.05) using the stat package in R (version 3.6.3). The top 10 IGF2BP1-correlated genes, proteins, and lncRNAs were visualized with R ggplot2 (version 3.3.3), respectively. We also zbscreened correlated proteins from the QExact platform proteome datasets with LinkedOmics (Vasaikar et al., 2018) which is a unique online analytical platform that provides comprehensive multi-omics data analysis using default settings, and visualized them with heatmaps.

### 2.8 Statistical methods

We employed the R package to perform statistical analysis. Differences between groups were compared using the Student’s t-test, paired t-test, or nonparametric Wilcoxon rank-sum test, as appropriate. We computed correlations in R using cor with options for Pearson or Spearman correlation tests, as appropriate. Kaplan-Meier plots were generated and log-rank tests were executed to identify significant differences between survival curves, and differences of *p* < 0.05 were considered statistically significant.

## 3 Results

### 3.1 Relationship between genetic alterations in insulin-like growth factor 2 mRNA binding protein 1 and survival and clinical attributes in the cBioportal dataset

**FIGURE 1:**
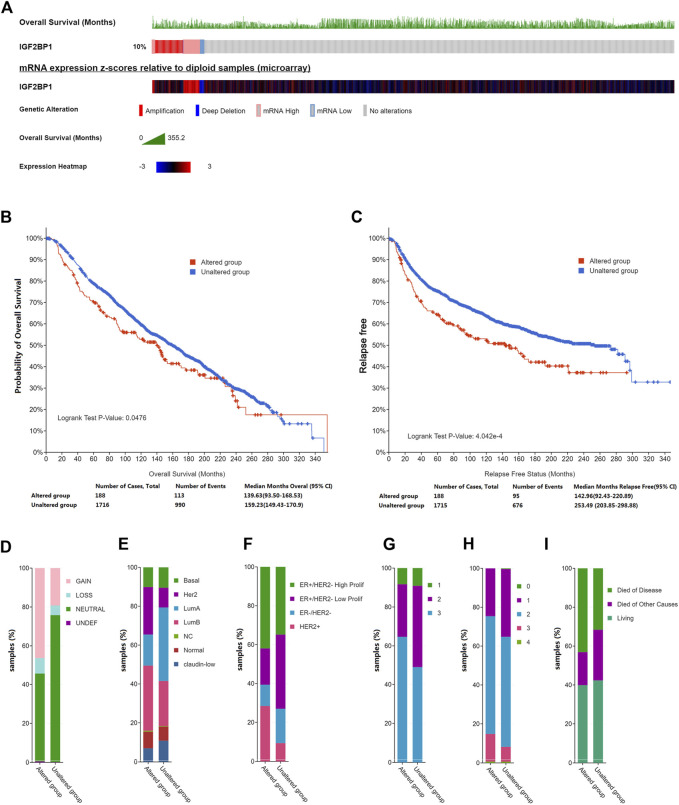
Genetic alterations in IGF2BP1 and their relation to clinical attributes by cBioportal. **(A)**, Genetic alterations and mRNA expression of IGF2BP1. **(B)** and **(C)**, The relationship between genetic alterations in IGF2BP1 and OS or RFS. **(D–I)**, The relationship between genetic alterations of IGF2BP1 and clinical attributes.

To elucidate the role of IGF2BP1 in breast cancer, we first interrogated large publicly available datasets from METABRIC using cBioPortal and analyzed genetic alterations in IGF2BP1 that included mRNA amplifaction, deletion or level changes and their correlation with clinical values. Using a dataset from the targeted sequences of 2,509 primary breast tumors and 548 matched normal controls (Curtis, et al., 2012; Pereira, et al., 2016), we analyzed IGF2BP1-gene alterations in 1904 patients/samples with available mutation and CNA data and observed a change for IGF2BP1 mRNA in breast cancer of 10% (188/1904). Amplification and elevated mRNA levels accounted for the majority of the genetic alterations in IGF2BP1 (Figure 1A). Genetically altered IGF2BP1 predicted a poor prognosis for both OS and relapse-free status (*p* < 0.05, Figures 1B,C), while alterations in IGF2BP1 demonstrated a robust relationship with clinical attributes. Specifically, the IGF2BP1 altered group exhibited a greater incidence of HER2 mRNA gain (Figure 1D), higher proportion of HER2 and luminal B subtypes by prediction analysis of microarray 50 (PAM50) (Figure 1E) or HER2 and ER+/HER2- with high proliferating subtypes by three genes classifiers (Figure 1F), a tendancy to manifest, grade-3 histology (Figure 1G), higher clinical tumor stages (Figure 1H), and an increased probability of disease-specific death (Figure 1I).

### 3.2 Insulin-like growth factor 2 mRNA binding protein 1 expression is augmented in breast cancer tissues using TCGA and gene expression omnibus databases

**FIGURE 2:**
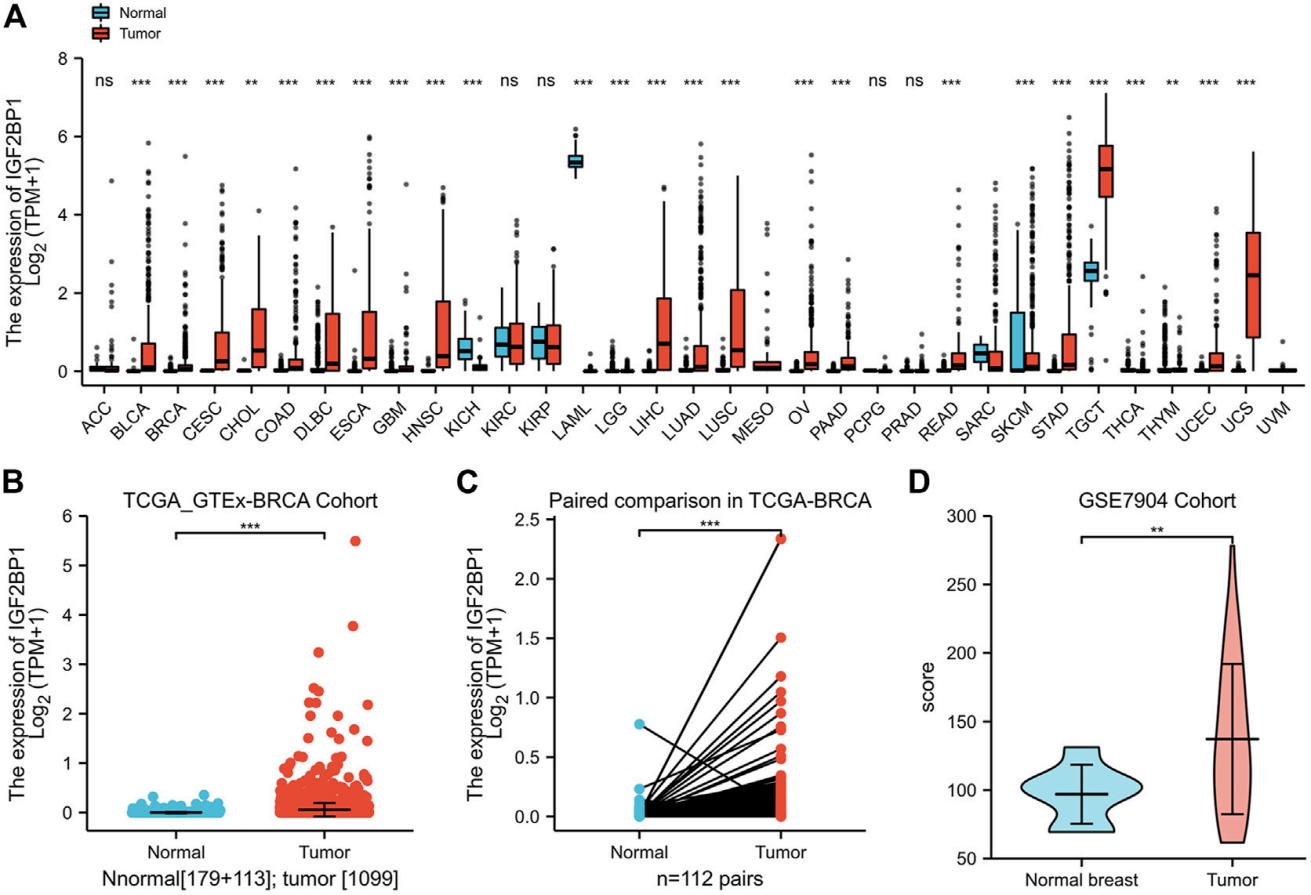
Expression of IGF2BP1 in various cancers and BRCA in particular. **(A)**, The expression of IGF2BP1 in various cancers. **(B)**, The expression of IGF2BP1 in TCGA-GTEx-BRCA cohort samples. **(C)**, The expression of IGF2BP1 in TCGA -BRCA paired samples. **(D)**, The expression of IGF2BP1 in the GSE7904 cohort. *p < 0.05; **p < 0.01; ***p <.001.

As amplification and elevated mRNA levels accounted for most of the genetic alterations of prognostic IGF2BP1, we consequently analyzed mRNA levels in a large patient cohort of 15,776 samples with 33 types of human cancer and 31 types of adjacent normal tissues in TCGA datasets and GTEx samples accessible through UCSC Xena. We ascertained IGF2BP1 was significantly upregulated in most cancer types as compared with the corresponding normal tissues, but not in adrenocortical carcinoma (ACC), kidney, renal clear cell carcinoma (KIRC), kidney renal papillary cell carcinoma (KIRP), pheochromocytoma and paraganglioma (PCPG), and prostate adenocarcinoma (PRAD) (Figure 2A). We specifically noted that IGF2BP1 was more highly expressed in BRCA tissues in both unmatched (tumor, *n* = 1,109 vs. adjacent and normal, *n* = 292) and paired (112 paired tumors vs. adjacent normal) comparisons of TCGA-BRCA datasets compared to normal tissues (Figures 2B,C). A majority of paired samples also exhibited upregulation, while a minute subset demonstrated downregulation of IGF2BP1 in cancer tissue (Figure 2C). Our analysis of gene expression data of the GSE7904 cohort (seven normals vs. 43 tumors) validated the overexpression of IGF2BP1 at the mRNA level (Figure 2D).

### 3.3 Insulin-like growth factor 2 mRNA binding protein 1 expression correlates with histologic and intrinsic subtyping of breast cancer

**FIGURE 3:**
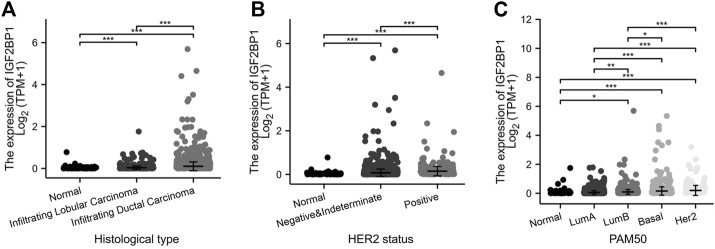
Relationship between IGF2BP1 expression and histologic and molecular features in TCGA-BRCA datasets. **(A)**, Relationship between IGF2BP1 expression and histologic types. **(B)**, Relationship between IGF2BP1 expression and HER2 status of breast cancer. **(C)**, Relationship between IGF2BP1 expression and PAM50 molecular subtypes of breast cancer. *p < 0.05; **p < 0.01; ***p <.001.

Using the expression and clinical data from the TCGA-BRCA dataset, we demonstrated that the expression of IGF2BP1 mRNA was markedly correlated with histologic type (Figure 3A), and that molecular subtypes of breast cancer were defined by HER2 status (Figure 3B) or PAM50 (Figure 3C). In addition to discriminating cancerous from normal cells, the mRNA levels of IGF2BP1 were found to be significantly increased in certain subgroups of breast cancer. For example, the highest level was found in invasive ductal carcinoma (IDC) (*n* = 772) when compared to invasive lobular carcinoma (ILC) (*n* = 205) and normal breast tissues (*n* = 111), as well as in the HER2-positive subgroup (*n* = 157) when compared to the HER2-negative group (*n* = 570) or normal breast tissues (*n* = 111). Our data revealed IGF2BP1 at differential levels among intrinsic subtypes of breast cancer (Normal, *n* = 40; LumA, *n* = 562; LumB, *n* = 204; Basal, *n* = 195; Her2, *n* = 82: Kruskal–Wallis test, *p* < 0.001), which raised the question whether IGF2BP1 was related to breast cancer prognosis.

### 3.4 Relationships of insulin-like growth factor 2 mRNA binding protein 1 expression with OS and disease-specific survival in breast carcinoma patients in TCGA database

**FIGURE 4:**
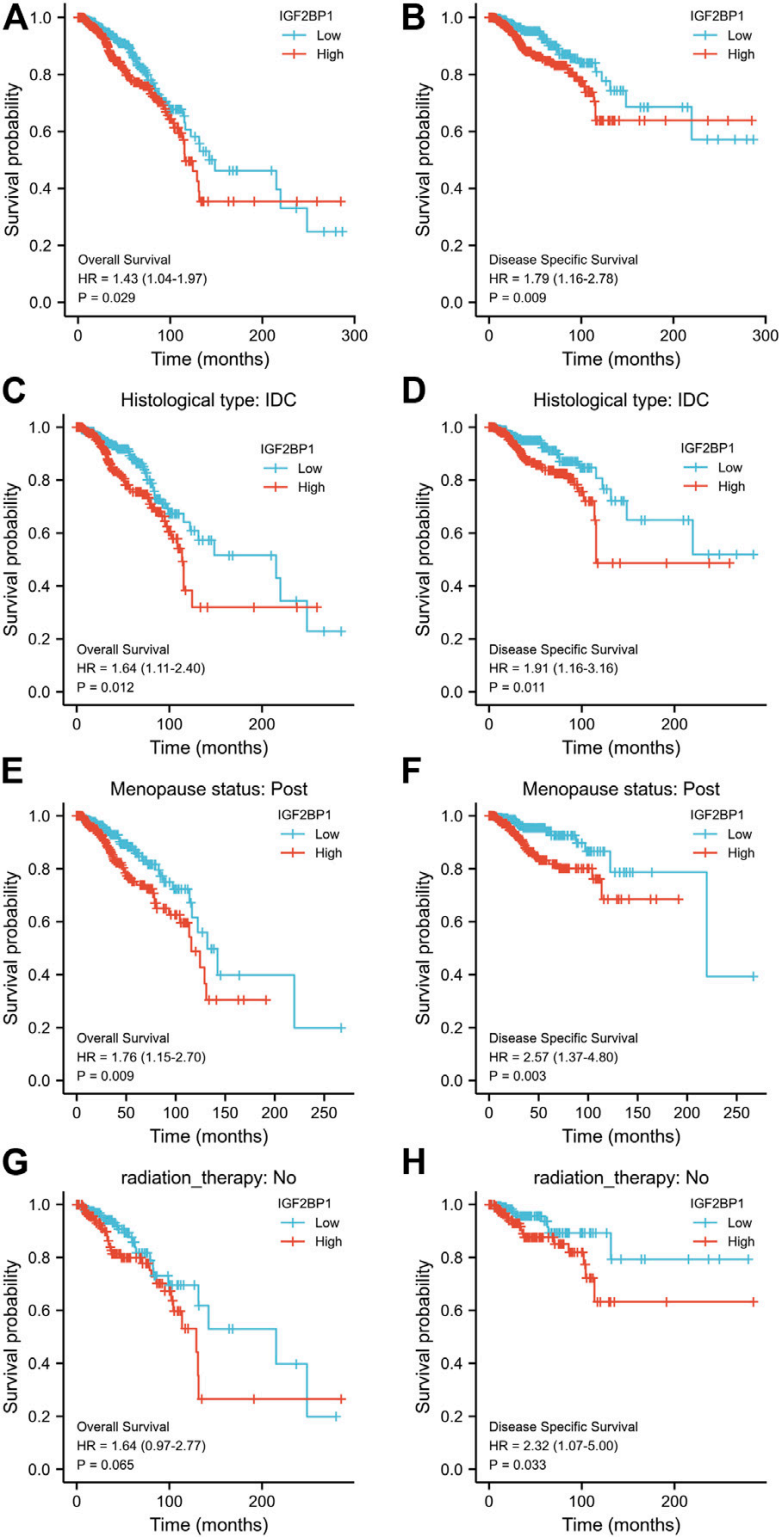
Relationship between IGF2BP1 expression and the prognosis of BRCA patients in TCGA database. **(A)** and **(B)**, Comparison of Kaplan–Meier curves for OS or DSS between high- and low-IGF2BP1 expressing groups. **(C−H)**, Compared Kaplan–Meier curves for OS or DSS between high- and low-IGF2BP1 expression in subgroups of IDC, post menopause or without radiation therapy, respectively.

We divided the cohorts into low or high-expression subgroups according to the median expression of IGF2BP1 mRNA to compare IGF2BP1 expression-related outcomes of the patients. Kaplan–Meier curves for OS and DSS revealed that BRCA patients with high IGF2BP1 expression possessed a generally worse prognosis than patients with low IGF2BP1 expression (*p* < 0.01, Figures 4A,B). Further analysis indicated that high IGF2BP1 expression argued an unfavorable prognosis for both OS and DSS in the subgroups of IDC and women in postmenopausal status (*p* < 0.01, Figures 4C–F), while it was prognostic for DSS but not for OS in the subgroup that did not undergo radiation therapy (Figures 4G,H). The analysis of other subgroups (including histologic ILC subgroup, peri- and pre-menopausal status, HER2 status, ER status, and PR status) achieved no significant prognostic relevance with respect to IGF2BP1 overexpression (data not shown). The 5- and 10-year OS rates in the database were higher among patients with low IGF2BP1 expression relative to those with high expression (85.95 ± 2.4% vs. 77.3 ± 2.8% and 58.2 ± 5.8% vs.46.1 ± 6.5%, respectively; *p* < 0.001).

### 3.5 Generation of nomogram model

**FIGURE 5:**
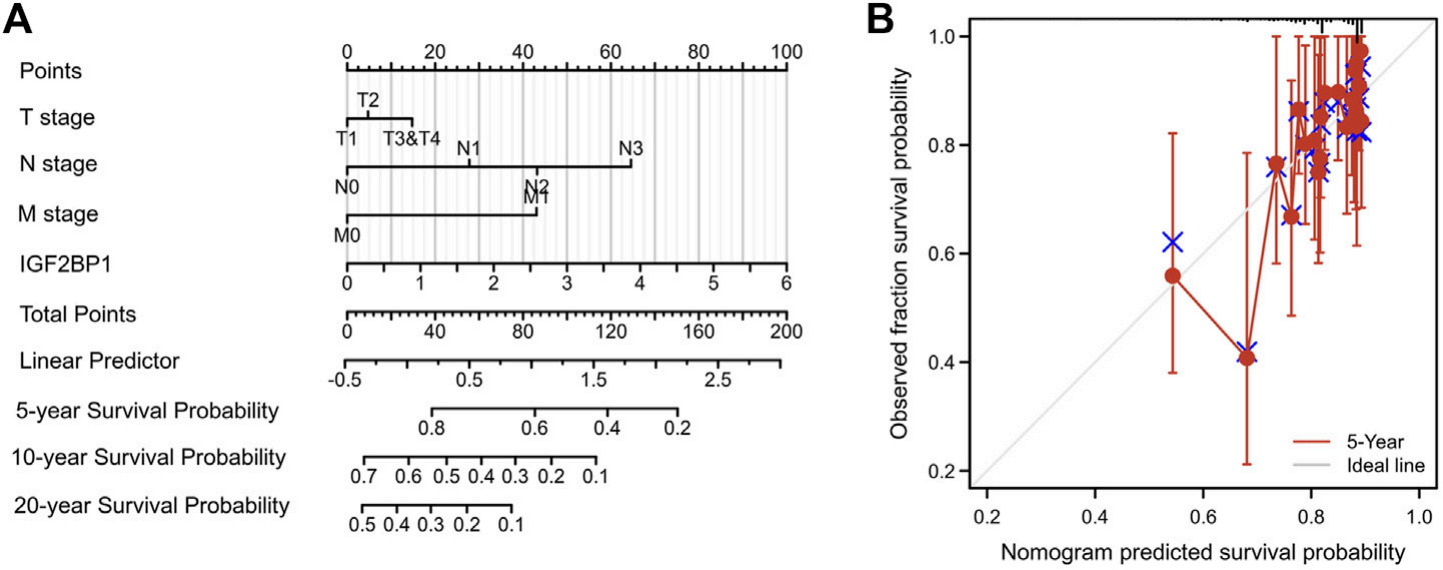
Nomogram model for predicting overall survival. **(A)**, Nomogram for predicting overall survival at 5 years, 10 years and 20 years. **(B)**, Nomogram calibration plot for five-year OS as a representative index.

Using survival data from 876 of 1,082 patient cohorts in the TCGA database, we further implemented uni- and multivariate survival analyses using Cox proportional hazards models for survival-related factors; and the results showed that T stage, N stage, and M stage of the tumor; PAM50 subtyping; and IGF2BP1 expression were predictors of OS upon univariate Cox analysis. Importantly, N stage, M stage, and IGF2BP1 expression were independent variables in our multivariate Cox analysis, with a concordance index of 0.694 (0.661–0.726) (Table 1). When we then constructed a nomogram for predicting overall survival at 5 years, 10 years, and 20 years based on these models (Figure 5A), we discern that the nomogram calibration plot showed acceptable agreement between nomogram-predicted and observed events (an example of five-year OS is depicted in Figure 5B.

**TABLE 1:** Results of Cox univariate and multivariate analyses.

Characteristics	Univariate analysis		Multivariate analysis	
	Hazard ratio (95% CI)	*p* value	Hazard ratio (95% CI)	*p* value
T stage				
T1	Reference			
T2	1.332 (0.887–1.999)	0.166	1.123 (0.697–1.810)	0.634
T3&T4	1.953 (1.221–3.123)	**0.005**	1.448 (0.799–2.624)	0.222
N stage				
N0	Reference			
N1	1.956 (1.329–2.879)	**<0.001**	1.767 (1.148–2.719)	**0.010**
N2	2.519 (1.482–4.281)	**<0.001**	2.245 (1.253–4.023)	**0.007**
N3	4.188 (2.316–7.574)	**<0.001**	3.467 (1.561–7.698)	**0.002**
M stage				
M0	Reference			
M1	4.254 (2.468–7.334)	**<0.001**	2.014 (1.025–3.961)	**0.042**
PAM50				
LumA	Reference			
LumB	1.663 (1.088–2.541)	**0.019**	1.400 (0.887–2.208)	0.148
Her2	2.261 (1.325–3.859)	**0.003**	1.719 (0.933–3.167)	0.082
Basal	1.285 (0.833–1.981)	0.257	1.413 (0.874–2.285)	0.158
IGF2BP1				
Low	Reference			
High	1.463 (1.060–2.018)	**0.021**	1.458 (1.003–2.119)	**0.048**

Bold values reprsents that the *p* value of less than 0.05 indicated statistical significance.

### 3.6 Functional-enrichment analysis of differentially expressed genes between high and low-insulin-like growth factor 2 mRNA binding protein 1 expression samples

**FIGURE 6:**
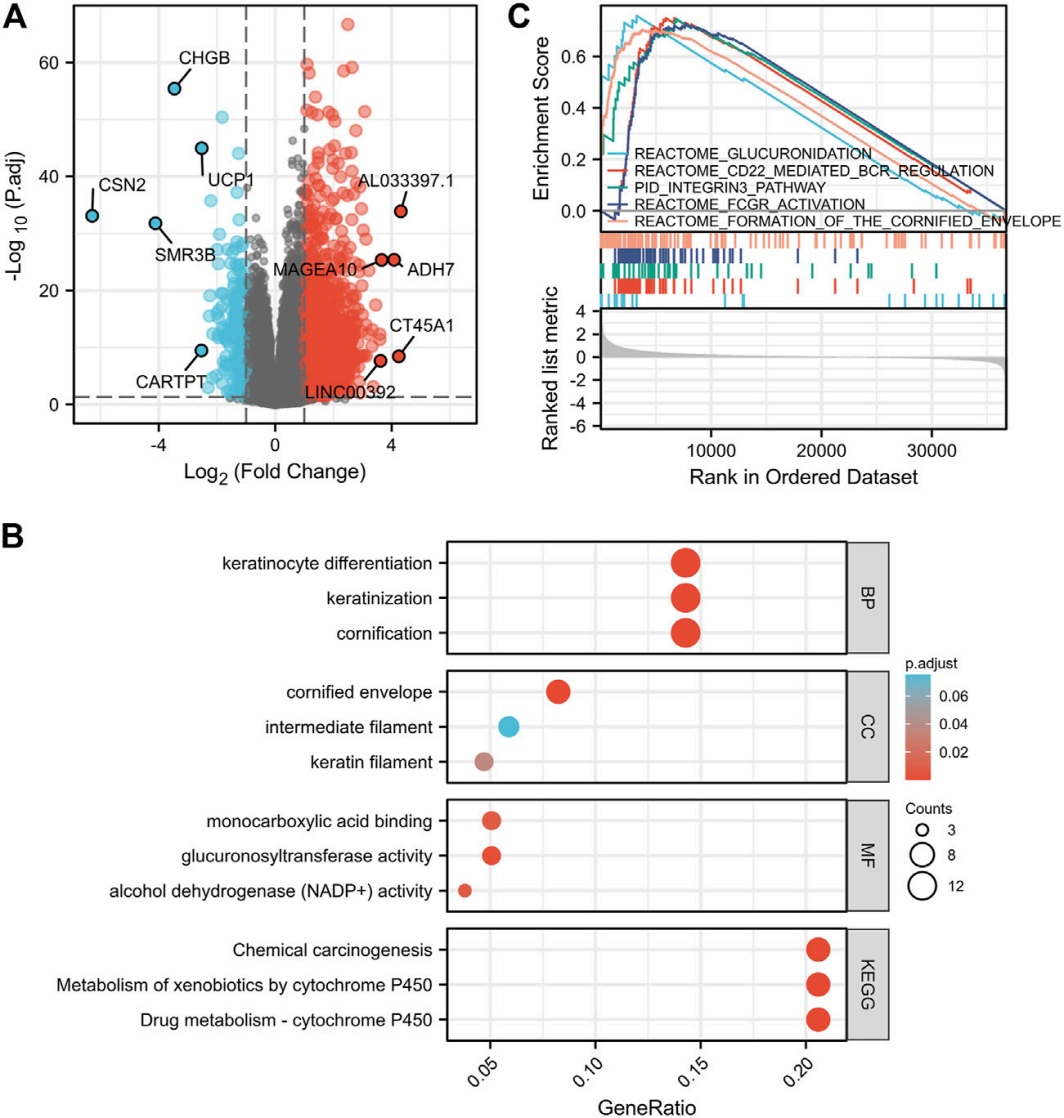
Pathway-enrichment analyses of DEGs between high- and low-IGF2BP1 expression samples. **(A)**, Volcano plot for the DEGs expression with the top five upregulated and five down-regulated genes displayed. **(B)**, Bubble diagram of the top enriched GO|KEGG pathway results for DEGs. **(C)**, GSEA of DEGs using the Reactome Pathway Database.

To explore the potential prognostic mechanisms underlying IGF2BP1 action, we analyzed DEGs in the high- and low-IGF2BP1 expression groups and identified a total of 2,405 DEGs of 56,493 genes, of which 2,199 genes were upregulated and 206 were downregulated. When filtering with |log2 fold-change| > 1.5, we identified 875 genes, of which 828 were upregulated and 47 downregulated, and 303 genes were identified when filtering with |log2 fold-change| > 2.0, with 290 upregulated and 13 downregulated (Supplementary Table S1). DEG expression is displayed in Figure 6A in the volcano plot encompassing the top five upregulated and five downregulated genes. Functional analysis of DEGs with a |log2 fold change|> 2.0 revealed 81 GO terms and 13 KEGG pathways at p. adj<0.05 and qvalue<0.2. The top GO terms were cornification, keratinization, keratinocyte differentiation, cornified envelope, keratin filament, glucuronosyltransferase activity, alcohol dehydrogenase (NADP+) activity, aldo-keto reductase (NADP) activity, and monocarboxylic acid binding for biological process (BP), molecular function (MF), and cellular component (CC). The top KEGG pathways were drug metabolism—cytochrome P450, metabolism of xenobiotics by cytochrome P450, and chemical carcinogenesis (Figure 6B, Supplementary Tables S2, S3). The GSEA-enrichment method and the Reactome pathway enrichment analysis for the DEG set exhibited some similar functionally relevant gene networks (Figure 6C). The 28 significantly enriched categories with FDR filtering (qvalue) < 0.25 and p. adjust<0.05 included formation of the cornified envelope (NES = 1.959; p. adjust = 0.020; FDR = 0.019), FCGR activation (NES = 1.942; p. adjust = 0.020; FDR = 0.019), CD22-mediated BCR regulation (NES = 1.979; p. adjust = 0.020; FDR = 0.019), integrin cell surface interactions (NES = 1.844; p. adjust = 0.020; FDR = 0.019), glucuronidation (NES = 1.792; p. adjust = 0.040; FDR = 0.038), and keratinization (NES = 1.788; p. adjust = 0.020; FDR = 0.019) (complete GSEA results are provided in Supplementary Table S4).

### 3.7 Functional enrichment analysis of correlated genes between high and low-insulin-like growth factor 2 mRNA binding protein 1 expression samples

**FIGURE 7:**
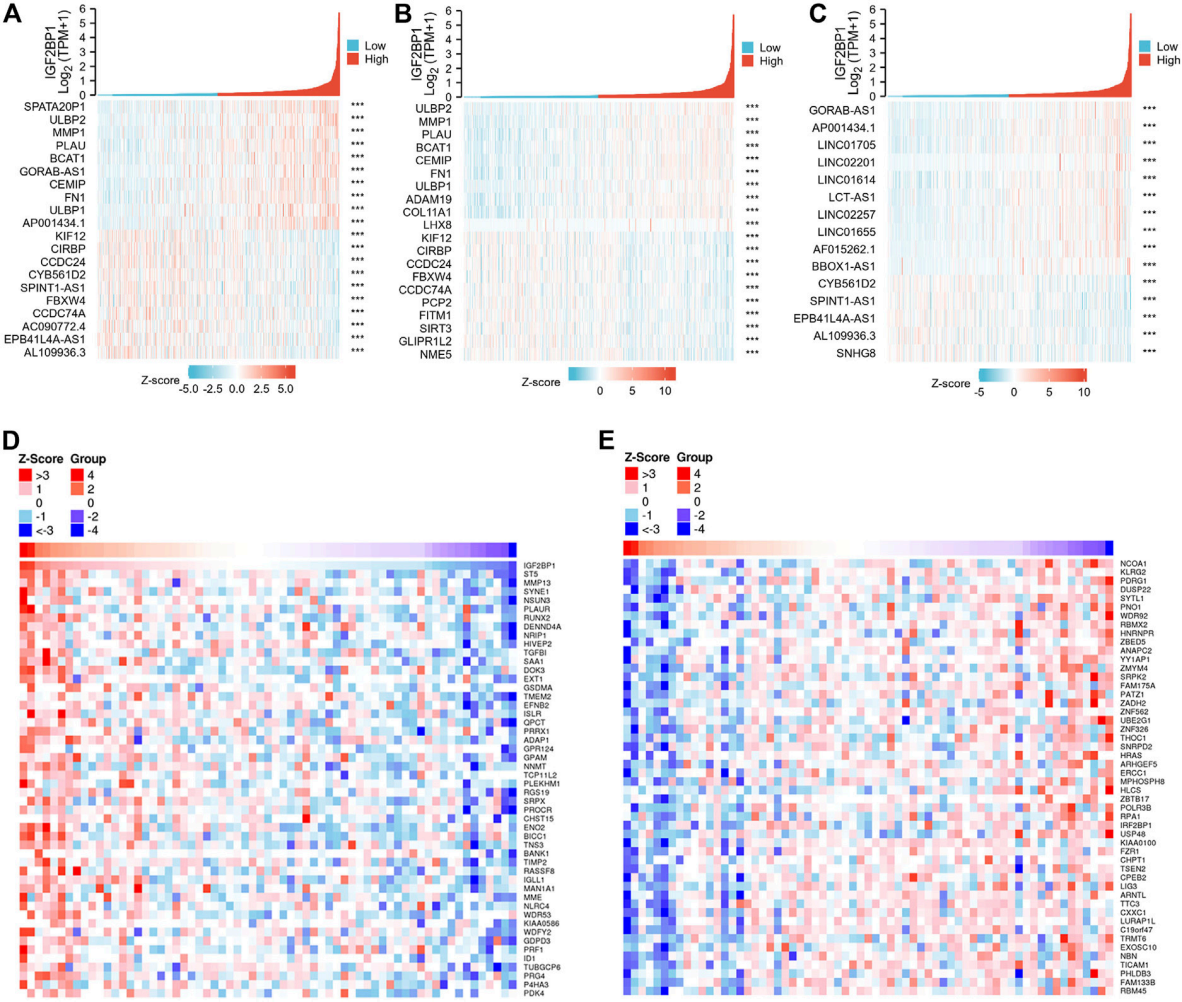
IGF2BP1-correlated genes and proteins from TCGA-BRCA databases and the LinkedOmics database. **(A–C)**, Heatmaps of top 10 positive and top 10 negative IGF2BP1-correlated genes, proteins and lncRNAs in TCGA-BRCA gene-expression database. **(D–E)**, Heatmaps of significantly positive and negatively IGF2BP1-correlated proteins in the proteome database using the LinkedOmics platform.

We then analyzed IGF2BP1-correlated genes and proteins from both the RNAseq dataset and proteome datasets to a screen for IGF2BP1 functional partners. Using the R package, we screened 134 genes that were positively correlated with IGF2BP1 mRNA expression from a total of 56,493 IDs, filtering them with Pearson’s correlation coefficient |cor| >0.3 and *p* < 0.05, and the top 10 genes, genes coding proteins, and genes for lncRNAs are displayed in our heatmaps (Figures 7A–C). LinkedOmics is a unique online analytical platform that provides comprehensive multi-omics data analysis (Vasaikar, et al., 2018), and we employed this platform to analyze IGF2BP1 protein partners using proteome datasets from 105 patients. We uncovered 1712 IGF2BP1-correlated proteins from 9,733 entries, and the most positively and negatively correlated proteins are displayed in our heatmaps (Figures 7D,E). We will in future investigations analyze the correlated genes, proteins, and lncRNAs using an interaction network and hub genes, and validate them in clinical samples.

## 4 Discussion

IGF2BP1 is principally expressed in embryos and in cancerous cells in contrast with comparatively lower or negligible levels in adult normal tissues, reflecting an ideal biomarker for disease. The prognostic overexpression of IGF2BP1 has been reported in over 16 cancers, whereas the overexpression of IGF2BP1 in colon (Hamilton et al., 2015) and breast cancer (Wang, et al., 2016) relative to normal tissues remains controversial. Breast cancer is a highly heterogeneous disease that shows substantial variations in molecular and clinical characteristics, and the discrepancy among studies regarding IGF2BP1 expression in breast cancer might be due to this sample heterogeneity (Huang et al., 2018b) that extends from histologic heterogeneity to subtypes, cellular, and even molecular heterogeneity; these disparities include metastatic status differences, cell types of different origins, phenotypic-functional heterogeneity, genomic discordances, expression-pattern changes, or genetic variations in certain molecules themselves during cancer development. For example, significantly augmented promoter methylation of IGF2BP1 leads to more common silencing events with respect to IGF2BP1 in metastatic breast tumor cells compared to methylation observed in non-metastatic breast tumor cells (Gu, et al., 2012). Moreover, IGF2BP1 was reported to be as a conserved ‘oncogenic’ m^6^A (N6-methylation of adenosine)-reader and could enhance mRNAs stability and translation through m^6^A modification (Huang et al., 2018a; Hao et al., 2020). Therefore, it is crucial to elucidate the detailed expression patterns of IGF2BP1 in heterogeneous breast cancer.

In the present study, we demonstrated genetic alterations (particularly amplification and overall upregulation) of IGF2BP1 in invasive breast cancer using large and publicly available datasets from METABRIC, TCGA, and GEO. We also determined that elevated IGF2BP1 mRNA expression was related to histologic and molecular subtypes, especially HER2 status in breast cancer a result not as controversial as that noted by others in the literature, and we found a very small subset of significantly downregulated IGF2BP1 in breast cancer tissues in our paired analysis. What drives the heightened expression of IGF2BP1 in the HER2-positive subtype is unknown, and the discrepancy between relative upregulation and downregulation of IGF2BP1 in paired analyses requires the elucidation of large and deep data mining. The clarification of these underlying mechanisms will facilitate the personalization of subtyping and treatment.

IGF2BP1 is functionally considered to promote tumor growth and invasion *via* the transport of certain mRNAs that play essential roles in embryogenesis, carcinogenesis, and chemo-resistance by affecting mRNA stability, translatability, or localization within most cancers (Huang et al., 2018b). IGF2BP1’s potential promotion of tumor invasiveness and progression in breast cancer, however, remains debatable. We also herein confirmed IGF2BP1 to be a promising prognostic indicator in overall invasive breast cancer and in certain subgroups, including histologic types of IDC and postmenopausal subgroups and patients who did not undergo radiotherapy. We additional analyzed the prognostic relevance of IGF2BP1 in breast cancer by applying enrichment analysis of DEGs, and our data revealed the involvement of pathways such as glucuronidation, cytochrome P450 (CYP) enzyme-related drug metabolism, cornification, and keratinization. Glucuronidation is a drug clearance and resistance mechanism (Mazerska et al., 2016) and cytochrome P450 (CYP) enzymes are responsible for the biotransformation of drugs involved in drug interactions and therapeutic efficacy (Stipp and Acco, 2021) in many diseases. In addition, keratinization and cornified envelope formation associated with proliferation and differentiation of keratinocytes and their progenitor cells (e.g., multipotent stem cells) remain generally stable during carcinogenesis and are correlated with disease aggressiveness and clinical outcomes (Choi et al., 2014; Blommel et al., 2020). We thereby hypothesized that an IGF2BP1-related unfavorable prognosis might be (at least partially) attributable to cellular differentiation as well as acquired drug resistance by altered drug biotransformation and clearance. We also executed correlation analysis using proteome datasets by mining the functional partners of IGF2BP1, as it has been shown that MMP family members were downregulated by IGF2BP1 silencing in ovarian carcinoma, thus reducing the invasive nature of tumor (Davidson et al., 2014). We thus plan to implement a systematic and exhaustive study as well as an experimental validation of the underlying mechanisms with respect to prognostic IGF2BP1 in breast cancer, with particular emphasis on hub genes in IGF2BP1 regulatory networks.

There were several limitations to the present study. First limitation was the lack of validation of the relative expression of IGF2BP1, its prognostic relevance in breast cancer, and the predictive capability of our established nomogram using external datasets such as large, multicenter cohorts. Second, the nomogram we implemented necessitates improvement to satisfy the requirement for convenience and accuracy using routine clinical data and laboratory tests such as the assessment of immune status of lymphocyte subsets. Finally, hub genes in IGF2BP1-targeted mRNA networks need to be identified, and our hypothesis of IGF2BP1 functional pathways based on enrichment analysis of DEGs and genetic correlation remains to be tested experimentally. The detailed molecular mechanisms by which IGF2BP1 regulates target mRNAs or vice versa warrants future investigation.

In summary, our results indicated that both genetic alterations in IGF2BP1 and increased levels of IGF2BP1 mRNA and protein predict a poor patient prognosis in BRCA patients. The findings will represent a potential therapeutic target for the treatment of BRCA.

## Data Availability

The datasets presented in this study can be found in online repositories. The names of the repository/repositories and accession number(s) can be found in the article/Supplementary Material.
