# Supplementary material for: Elevated insulin-like growth factor 2 mRNA binding protein 1 levels predict a poor prognosis in patients with breast carcinoma using an integrated multi-omics data analysis
Source: Front Genet. 2022 Aug 24;13:994003. doi: 10.3389/fgene.2022.994003 (PMC9448930; doi:10.3389/fgene.2022.994003)
Supplement: Supplementary file 1 [file DataSheet1.docx]

**Supplementary Materials**

**Table S1 Summary for DEGs between IGF2BP1 high and low expression groups**

| Filters | Number of genes | | |
| --- | --- | --- | --- |
|  | Total | IGF2BP1 high expression | IGF2BP1 low expression |
| Total IDs | 41245 |  |  |
| \|log2(FC)\|>1&p.adj<0.05 | 2405 | 2199 | 206 |
| \|log2(FC)\|>1.5&p.adj<0.05 | 875 | 828 | 47 |
| \|log2(FC)\|>2 & p.adj<0.05 | 303 | 290 | 13 |

**Table S2 Summary for GO|KEGG enrichment of DEGs between IGF2BP1 high and low expression groups**

| Filters | BP | CC | MF | KEGG |
| --- | --- | --- | --- | --- |
| p.adj<0.1&qvalue<0.2 | 85 | 5 | 16 | 14 |
| p.adj<0.05&qvalue<0.2 | 64 | 3 | 14 | 13 |
|  |  |  |  |  |

**Table S3 TOP Terms in GO|KEGG enrichment of DEGs between IGF2BP1 high and low expression groups**

| ONTOLOGY | | ID | Description | GeneRatio | BgRatio | | p.adjust | | qvalue | |  |
| --- | --- | --- | --- | --- | --- | --- | --- | --- | --- | --- | --- |
| BP | | GO:0070268 | cornification | 12/84 | 112/18670 | | 1.04e-10 | | 8.85e-11 | |  |
| BP | | GO:0031424 | keratinization | 12/84 | 224/18670 | | 1.94e-07 | | 1.66e-07 | |  |
| BP | | GO:0030216 | keratinocyte differentiation | 12/84 | 305/18670 | | 4.27e-06 | | 3.63e-06 | |  |
| BP | | GO:0009913 | epidermal cell differentiation | 12/84 | 358/18670 | | 1.87e-05 | | 1.59e-05 | |  |
| BP | | GO:0052697 | xenobiotic glucuronidation | 4/84 | 11/18670 | | 2.73e-05 | | 2.32e-05 | |  |
| CC | | GO:0001533 | cornified envelope | 7/85 | 65/19717 | | 1.19e-06 | | 1.13e-06 | |  |
| CC | | GO:0045095 | keratin filament | 4/85 | 95/19717 | | 0.037 | | 0.035 | |  |
| CC | | GO:0005882 | intermediate filament | 5/85 | 214/19717 | | 0.075 | | 0.072 | |  |
| MF | | GO:0015020 | glucuronosyltransferase activity | 4/79 | 33/17697 | | 0.002 | | 0.002 | |  |
| MF | | GO:0008106 | alcohol dehydrogenase (NADP+) activity | 3/79 | 21/17697 | | 0.008 | | 0.007 | |  |
| MF | | GO:0033293 | monocarboxylic acid binding | 4/79 | 64/17697 | | 0.008 | | 0.007 | |  |
| MF | | GO:0004033 | aldo-keto reductase (NADP) activity | 3/79 | 26/17697 | | 0.008 | | 0.007 | |  |
| MF | | GO:0005501 | retinoid binding | 3/79 | 35/17697 | | 0.013 | | 0.011 | |  |
| KEGG | | hsa00982 | Drug metabolism - cytochrome P450 | 7/34 | 71/8076 | | 9.38e-07 | | 7.63e-07 | |  |
| KEGG | hsa00980 | | Metabolism of xenobiotics by cytochrome P450 | 7/34 | | 77/8076 | 9.38e-07 | | 7.63e-07 | |  |
| KEGG | hsa05204 | | Chemical carcinogenesis | 7/34 | | 82/8076 | | 9.74e-07 | | 7.92e-07 | |
| KEGG | hsa00040 | | Pentose and glucuronate interconversions | 5/34 | | 34/8076 | | 4.90e-06 | | 3.98e-06 | |

**Table S4 Significant enriched reactome pathways by GSEA analysis for the DEG set IGF2BP1 high and low expression groups**

| ID | setSize | NES | p.adj | FDR | core_enrichment |
| --- | --- | --- | --- | --- | --- |
| REACTOME_EXTRACELLULAR_MATRIX_ORGANIZATION | 300 | 1.54892183 | 0.02036847 | 0.01908628 | FGG/FGA/MMP1/FGB/MMP13/MMP20/DSPP/ACAN/COL11A1/CAPNS2/MMP8/HAPLN1/MMP12/MMP10/DMP1/COL10A1/ITGB6/FN1/SPP1/MMP7/COL22A1/ITGA11/COL11A2/FBN3/IBSP/COL6A5/COL12A1/MFAP5/ADAM19/COL8A1/COL26A1/MMP11/ADAMTS2/CEACAM6/TLL2/COL5A1/LAMC2/FBN1/LOXL2/MMP9/FBN2/LOX/CAPN6/NID2/COL5A3/COL5A2/COMP/MMP14/ASPN/COL9A1/PLOD2/SDC1/ADAM12/ADAMTS14/VCAN/CTSK/COL1A2/COL13A1/LUM/KLK7/VCAM1/COL8A2/PLG/BMP1/COL24A1/PXDN/ADAMTS16/COL3A1/COL1A1/CTSV/MFAP2/MATN4/BMP7/ITGA5/COL6A3/ADAM8/CEACAM8/BGN/PRKCA/LTBP1/ICAM1/COL4A4/COL6A2/TGFB2/TIMP2/DDR2/SPARC/COL6A1/CAPN14/SERPINH1/NID1/EMILIN1/CTRB1/LAMA4/MMP3/P4HA3/EMILIN2/ITGA4/COL4A1/TLL1/ITGB1/P3H1/COL19A1/CTSS/ITGAV/CTSB/ITGAM/COL7A1/COL4A2/CTSL/ITGB3/SCUBE3/MMP2/P4HA2/ITGB2/ITGAX/ACTN1/CDH1/TNC/ADAM9/COLGALT1/DCN/FBLN2/PLOD1/SH3PXD2A/COL4A3/HSPG2/COL15A1/LAMC1/ITGA1/LAMB1/THBS1/COL9A3 |
| NABA_CORE_MATRISOME | 274 | 1.44220039 | 0.02036847 | 0.01908628 | FGG/FGA/ZP2/FGB/AMELX/ZP4/EPYC/DSPP/ACAN/COL11A1/RSPO2/HAPLN1/IGFBPL1/DMP1/COL10A1/FN1/SPP1/COL22A1/FNDC1/COL11A2/TGFBI/DMBT1/FBN3/TINAG/IBSP/ZPLD1/COL6A5/FGL1/COL12A1/SPOCK1/MFAP5/ZP1/COL8A1/COL26A1/MEPE/THBS2/COL5A1/LAMC2/FBN1/FBN2/OTOG/NID2/COL5A3/COL5A2/COMP/VWA3B/MXRA5/ASPN/COL9A1/POSTN/TSPEAR/VCAN/NYX/RSPO4/SRPX2/COL1A2/COL13A1/LUM/COL8A2/CCN4/COL24A1/AEBP1/CTHRC1/PXDN/OMD/COL3A1/COL1A1/MFAP2/MATN4/COL6A3/LGI2/NTNG2/BGN/LTBP1/COL4A4/COL6A2/OIT3/TECTB/COCH/SPARC/COL6A1/HAPLN3/FNDC7/NID1/EMILIN1/LAMA4/CCN5/HMCN1/EDIL3/EMILIN2/COL4A1/AMBN/COL19A1/MFGE8/COL7A1/COL4A2/TNFAIP6/ELSPBP1/TNC/FRAS1/ECM2/DCN/FBLN2/COL4A3/HSPG2/COL15A1/LAMC1/SPON1/LAMB1/THBS1/COL9A3/LAMB4/PODNL1/PRG4 |
| REACTOME_KERATINIZATION | 216 | 1.78820276 | 0.02036847 | 0.01908628 | SPRR2A/SPRR3/KRT13/KRT20/SPRR1B/KRT6A/KRT4/SPRR1A/KRT75/IVL/RPTN/SPRR2E/KRT16/LCE3D/SPRR2D/KRT81/KRT6C/DSG3/KRT9/KRT34/KRT79/KRT86/KRT78/KRTAP2-3/KRT12/KRT3/DSG4/KRT76/KRT83/SPINK6/KRTAP1-1/KLK5/SPRR2F/KRT85/FLG/CASP14/SPRR2B/KRT33B/TCHH/KRTAP1-5/KRT82/KLK14/LCE5A/KRT25/LCE1B/KRT6B/KRT24/KRT72/KRT80/CSTA/KRT84/KRT74/KRT2/KRTAP10-2/KLK8/KRTAP4-1/KRT26/KRTAP5-6/KRT40/KRT39/KRT73/LIPK/KRT23/KRTAP19-1/DSC3/SPRR2G/KRT71/LCE2C/KRTAP5-2/KRT28/SPINK5/KRTAP5-4 |
| REACTOME_IMMUNOREGULATORY_INTERACTIONS_BETWEEN_A_LYMPHOID_AND_A_NON_LYMPHOID_CELL | 186 | 1.61131085 | 0.02036847 | 0.01908628 | TREM1/ULBP1/ULBP3/IGKV1-5/NCR2/IGHV3-23/IGLV3-21/IGKV1-12/IGHV3-33/IGKV2D-40/IGKV4-1/IGHV3-30/IGHV1-2/IGHV4-34/IGKV2D-28/IGKV1-16/IGLV2-23/IGLV3-19/IGLV1-44/IGKV3-15/IGLV1-40/IGHV3-11/LILRA6/IGHV4-39/IGLV2-11/IGHV1-69/COL1A2/IGKV3-11/VCAM1/IGHV3-13/IGLV6-57/IGHV2-5/IGLV3-27/IGKV1D-39/IGHV3-48/IGKV1-17/IGHV4-59/IGKV1-33/IGLV1-47/COL3A1/COL1A1/IGLV2-14/FCGR3A/ICAM1/RAET1E/IGLV3-25/IGKV2-30/LILRB4/CD300E/IGHV2-70/OSCAR/PVR/IGHV3-53/IGKV1D-16/SIGLEC7/IGKV1D-12/TREML4/ITGA4/ITGB1/IGHV3-7/SIGLEC9/CD300LD/FCGR1A/SIGLEC1/SLAMF7/IGKV3D-20/LAIR1/ITGB2/CD300C/IGHV1-46/CD1A/IGKV3-20/LILRB3/SIGLEC12/CDH1/IGKV1D-33/KLRD1/LILRB5/TRAV29DV5/PILRA/CD300LF/LILRB1/IGLC3/LILRB2/IGLV1-51/CD99 |
| REACTOME_CELL_SURFACE_INTERACTIONS_AT_THE_VASCULAR_WALL | 194 | 1.70300119 | 0.02036847 | 0.01908628 | MMP1/CD177/TREM1/FN1/IGKV1-5/IGHV3-23/L1CAM/CEACAM5/IGLV3-21/IGKV1-12/IGHV3-33/GRB7/IGKV2D-40/PF4V1/IGKV4-1/IGHV3-30/CEACAM6/PSG1/PSG3/IGHV1-2/IGHV4-34/IGKV2D-28/IGKV1-16/IGLV2-23/IGLV3-19/IGLV1-44/PSG11/IGKV3-15/SDC1/IGLV1-40/IGHV3-11/CD84/IGHV4-39/PSG5/PSG2/IGLV2-11/IGHV1-69/OLR1/COL1A2/IGKV3-11/IGHV3-13/IGLV6-57/IGHV2-5/IGLV3-27/IGKV1D-39/IGHV3-48/IGKV1-17/IGHV4-59/SLC16A1/IGKV1-33/IGLV1-47/COL1A1/IGLV2-14/GYPB/ITGA5/SLC7A7/CEACAM8/ANGPT1/PSG8/IGLL1/IGLV3-25/IGKV2-30/LYN/IGHV2-70/IGHV3-53/IGKV1D-16/FCAMR/IGKV1D-12/SLC16A3/ITGA4/ITGB1/IGHV3-7/ITGAV/PSG9/ITGAM/TNFRSF10D/ITGB3/SLC7A5/IGKV3D-20/IGHM/PROC/ITGB2/IGHV1-46/SLC7A6/IGKV3-20/ITGAX/PSG7/IGKV1D-33/FCER1G/MERTK/VPREB1/IGLC3/IGLV1-51/SLC7A11/CD99/PSG4/ANGPT2/YES1/CXADR/IGLV3-1/SDC2/SIRPA/GLG1 |
| REACTOME_FCGAMMA_RECEPTOR_FCGR_DEPENDENT_PHAGOCYTOSIS | 143 | 1.55287661 | 0.02036847 | 0.01908628 | IGKV1-5/IGHV3-23/IGLV3-21/IGKV1-12/IGHV3-33/IGKV2D-40/IGKV4-1/IGHV3-30/IGHG1/PLPP4/IGHV1-2/IGHV4-34/IGKV2D-28/IGKV1-16/IGLV2-23/IGLV3-19/IGLV1-44/IGKV3-15/IGLV1-40/IGHV3-11/IGHV4-39/IGLV2-11/IGHV1-69/IGKV3-11/IGHV3-13/IGLV6-57/IGHV2-5/IGLV3-27/IGKV1D-39/IGHV3-48/MYO10/IGKV1-17/IGHV4-59/IGKV1-33/IGLV1-47/IGLV2-14/FCGR3A/IGLV3-25/IGKV2-30/LYN/IGHV2-70/IGHV3-53/IGKV1D-16/IGKV1D-12/IGHV3-7/FCGR1A/IGKV3D-20/IGHV1-46/IGKV3-20/IGKV1D-33/HCK/ACTR3/WIPF2/NCKAP1L/IGLC3/IGLV1-51/WIPF1/MYH9/YES1/IGHG4/ACTR2/HSP90AA1/FCGR2A/IGLV3-1/MYH2/IGLC2/ITPR3/BTK/ARPC2/MYO5A/IGHG2/ARPC1B/VAV1/WASF3/FGR/NCKAP1/LIMK1/ACTB/MYO9B/IGKV1-39/PLD1/ABI1/FYN/PIK3CA/CD3G/SRC |
| REACTOME_FCERI_MEDIATED_NF_KB_ACTIVATION | 136 | 1.58623515 | 0.02036847 | 0.01908628 | IGKV1-5/IGHV3-23/IGLV3-21/IGKV1-12/IGHV3-33/IGKV2D-40/IGKV4-1/IGHV3-30/IGHV1-2/IGHV4-34/IGKV2D-28/IGKV1-16/IGLV2-23/IGLV3-19/IGLV1-44/IGKV3-15/IGLV1-40/IGHV3-11/IGHV4-39/IGLV2-11/IGHV1-69/IGKV3-11/IGHV3-13/IGLV6-57/IGHV2-5/IGLV3-27/IGKV1D-39/IGHV3-48/IGKV1-17/IGHV4-59/IGKV1-33/IGLV1-47/IGLV2-14/IGLV3-25/IGKV2-30/LYN/IGHV2-70/IGHV3-53/IGKV1D-16/IGKV1D-12/IGHV3-7/PSMD3/IGKV3D-20/IGHV1-46/IGKV3-20/IGKV1D-33/FCER1G/PSMD7/PSMA8/IGLC3/IGLV1-51 |
| REACTOME_DEGRADATION_OF_THE_EXTRACELLULAR_MATRIX | 140 | 1.55254269 | 0.02036847 | 0.01908628 | MMP1/MMP13/MMP20/ACAN/COL11A1/CAPNS2/MMP8/MMP12/MMP10/COL10A1/FN1/SPP1/MMP7/COL11A2/FBN3/COL6A5/COL12A1/COL8A1/COL26A1/MMP11/TLL2/COL5A1/LAMC2/FBN1/MMP9/FBN2/CAPN6/COL5A3/COL5A2/MMP14/COL9A1/CTSK/COL1A2/COL13A1/KLK7/COL8A2/PLG/BMP1/ADAMTS16/COL3A1/COL1A1/CTSV/COL6A3/ADAM8/COL4A4/COL6A2/TIMP2/COL6A1/CAPN14/NID1/CTRB1/MMP3/COL4A1/TLL1/COL19A1/CTSS/CTSB/COL7A1/COL4A2/CTSL/SCUBE3/MMP2/CDH1/ADAM9/DCN/COL4A3/HSPG2/COL15A1/LAMC1/LAMB1/COL9A3/MMP15/ADAM17 |
| REACTOME_FORMATION_OF_THE_CORNIFIED_ENVELOPE | 128 | 1.95913117 | 0.02036847 | 0.01908628 | SPRR2A/SPRR3/KRT13/KRT20/SPRR1B/KRT6A/KRT4/SPRR1A/KRT75/IVL/RPTN/SPRR2E/KRT16/LCE3D/SPRR2D/KRT81/KRT6C/DSG3/KRT9/KRT34/KRT79/KRT86/KRT78/KRT12/KRT3/DSG4/KRT76/KRT83/SPINK6/KLK5/SPRR2F/KRT85/FLG/CASP14/SPRR2B/KRT33B/TCHH/KRT82/KLK14/LCE5A/KRT25/LCE1B/KRT6B/KRT24/KRT72/KRT80/CSTA/KRT84/KRT74/KRT2/KLK8/KRT26/KRT40/KRT39/KRT73/LIPK/KRT23 |
| REACTOME_PARASITE_INFECTION | 116 | 1.72356666 | 0.02036847 | 0.01908628 | IGKV1-5/IGHV3-23/IGLV3-21/IGKV1-12/IGHV3-33/IGKV2D-40/IGKV4-1/IGHV3-30/IGHG1/IGHV1-2/IGHV4-34/IGKV2D-28/IGKV1-16/IGLV2-23/IGLV3-19/IGLV1-44/IGKV3-15/IGLV1-40/IGHV3-11/IGHV4-39/IGLV2-11/IGHV1-69/IGKV3-11/IGHV3-13/IGLV6-57/IGHV2-5/IGLV3-27/IGKV1D-39/IGHV3-48/MYO10/IGKV1-17/IGHV4-59/IGKV1-33/IGLV1-47/IGLV2-14/FCGR3A/IGLV3-25/IGKV2-30/LYN/IGHV2-70/IGHV3-53/IGKV1D-16/IGKV1D-12/IGHV3-7/IGKV3D-20/IGHV1-46/IGKV3-20/IGKV1D-33/HCK/ACTR3/WIPF2/NCKAP1L/IGLC3/IGLV1-51/WIPF1/MYH9/YES1/IGHG4/ACTR2/IGLV3-1/MYH2/IGLC2/BTK/ARPC2/MYO5A/IGHG2/ARPC1B/VAV1/WASF3/FGR/NCKAP1/ACTB/MYO9B/IGKV1-39/ABI1/FYN/CD3G/SRC |
| REACTOME_FCGR3A_MEDIATED_IL10_SYNTHESIS | 95 | 1.70743443 | 0.02036847 | 0.01908628 | IGKV1-5/IGHV3-23/IGLV3-21/IGKV1-12/IGHV3-33/IGKV2D-40/IGKV4-1/IGHV3-30/IGHG1/IGHV1-2/IGHV4-34/IGKV2D-28/IGKV1-16/IGLV2-23/IGLV3-19/IGLV1-44/IGKV3-15/ADCY7/IGLV1-40/IGHV3-11/IGHV4-39/IGLV2-11/IGHV1-69/IGKV3-11/IGHV3-13/IGLV6-57/IGHV2-5/IGLV3-27/IGKV1D-39/IGHV3-48/IGKV1-17/IGHV4-59/IGKV1-33/IGLV1-47/IGLV2-14/FCGR3A/IGLV3-25/IGKV2-30/LYN/IGHV2-70/IGHV3-53/IGKV1D-16/IGKV1D-12/IGHV3-7/FCGR1A/IGKV3D-20/IL10/IGHV1-46/IGKV3-20/IGKV1D-33/HCK/PRKX/IGLC3/IGLV1-51/YES1/IGHG4/FCGR2A/IGLV3-1 |
| REACTOME_COLLAGEN_FORMATION | 90 | 1.74185675 | 0.02036847 | 0.01908628 | MMP13/MMP20/COL11A1/COL10A1/MMP7/COL22A1/COL11A2/COL6A5/COL12A1/COL8A1/COL26A1/ADAMTS2/TLL2/COL5A1/LAMC2/LOXL2/MMP9/LOX/COL5A3/COL5A2/COL9A1/PLOD2/ADAMTS14/COL1A2/COL13A1/COL8A2/BMP1/COL24A1/PXDN/COL3A1/COL1A1/CTSV/COL6A3/COL4A4/COL6A2/COL6A1/SERPINH1/MMP3/P4HA3/COL4A1/TLL1/P3H1/COL19A1/CTSS/CTSB/COL7A1/COL4A2/CTSL/P4HA2/COLGALT1/PLOD1/COL4A3/COL15A1/COL9A3/COL20A1/PCOLCE/PCOLCE2/P4HA1/COL18A1 |
| REACTOME_FCERI_MEDIATED_MAPK_ACTIVATION | 87 | 1.7097274 | 0.02036847 | 0.01908628 | IGKV1-5/IGHV3-23/IGLV3-21/IGKV1-12/IGHV3-33/IGKV2D-40/IGKV4-1/IGHV3-30/IGHV1-2/IGHV4-34/IGKV2D-28/IGKV1-16/IGLV2-23/IGLV3-19/IGLV1-44/IGKV3-15/IGLV1-40/IGHV3-11/IGHV4-39/IGLV2-11/IGHV1-69/IGKV3-11/IGHV3-13/IGLV6-57/IGHV2-5/IGLV3-27/IGKV1D-39/IGHV3-48/IGKV1-17/IGHV4-59/IGKV1-33/IGLV1-47/IGLV2-14/IGLV3-25/IGKV2-30/LYN/IGHV2-70/IGHV3-53/IGKV1D-16/IGKV1D-12/IGHV3-7/IGKV3D-20/IGHV1-46/IGKV3-20/IGKV1D-33/FCER1G/IGLC3/IGLV1-51 |
| REACTOME_ANTIGEN_ACTIVATES_B_CELL_RECEPTOR_BCR_LEADING_TO_GENERATION_OF_SECOND_MESSENGERS | 86 | 1.76327281 | 0.02036847 | 0.01908628 | IGKV1-5/IGHV3-23/IGLV3-21/IGKV1-12/IGHV3-33/IGKV2D-40/IGKV4-1/IGHV3-30/IGHV1-2/IGHV4-34/IGKV2D-28/IGKV1-16/IGLV2-23/IGLV3-19/IGLV1-44/IGKV3-15/IGLV1-40/IGHV3-11/IGHV4-39/IGLV2-11/IGHV1-69/IGKV3-11/IGHV3-13/IGLV6-57/IGHV2-5/IGLV3-27/IGKV1D-39/IGHV3-48/IGKV1-17/IGHV4-59/IGKV1-33/IGLV1-47/IGLV2-14/PIK3AP1/IGLV3-25/IGKV2-30/LYN/IGHV2-70/IGHV3-53/IGKV1D-16/DAPP1/IGKV1D-12/IGHV3-7/IGKV3D-20/IGHM/IGHV1-46/IGKV3-20/IGKV1D-33 |
| REACTOME_FCERI_MEDIATED_CA_2_MOBILIZATION | 86 | 1.74455603 | 0.02036847 | 0.01908628 | IGKV1-5/IGHV3-23/IGLV3-21/IGKV1-12/IGHV3-33/IGKV2D-40/IGKV4-1/IGHV3-30/IGHV1-2/IGHV4-34/IGKV2D-28/IGKV1-16/IGLV2-23/IGLV3-19/IGLV1-44/IGKV3-15/IGLV1-40/IGHV3-11/IGHV4-39/IGLV2-11/IGHV1-69/IGKV3-11/IGHV3-13/IGLV6-57/IGHV2-5/IGLV3-27/IGKV1D-39/IGHV3-48/IGKV1-17/IGHV4-59/IGKV1-33/IGLV1-47/IGLV2-14/IGLV3-25/IGKV2-30/LYN/IGHV2-70/IGHV3-53/IGKV1D-16/IGKV1D-12/IGHV3-7/IGKV3D-20/IGHV1-46/IGKV3-20/IGKV1D-33/FCER1G/IGLC3/IGLV1-51/NFATC3/IGLV3-1/LCP2/IGLC2/SOS1/ITPR3/BTK |
| REACTOME_INTEGRIN_CELL_SURFACE_INTERACTIONS | 84 | 1.84409835 | 0.02036847 | 0.01908628 | FGG/FGA/FGB/COL10A1/ITGB6/FN1/SPP1/ITGA11/IBSP/COL6A5/COL8A1/COL5A1/FBN1/COL5A3/COL5A2/COMP/COL9A1/COL1A2/COL13A1/LUM/VCAM1/COL8A2/COL3A1/COL1A1/ITGA5/COL6A3/ICAM1/COL4A4/COL6A2/COL6A1/ITGA4/COL4A1/ITGB1/ITGAV/ITGAM/COL7A1/COL4A2/ITGB3/ITGB2/ITGAX/CDH1/TNC/COL4A3/HSPG2/ITGA1/THBS1/COL9A3 |
| REACTOME_ROLE_OF_PHOSPHOLIPIDS_IN_PHAGOCYTOSIS | 82 | 1.76097839 | 0.02036847 | 0.01908628 | IGKV1-5/IGHV3-23/IGLV3-21/IGKV1-12/IGHV3-33/IGKV2D-40/IGKV4-1/IGHV3-30/IGHG1/PLPP4/IGHV1-2/IGHV4-34/IGKV2D-28/IGKV1-16/IGLV2-23/IGLV3-19/IGLV1-44/IGKV3-15/IGLV1-40/IGHV3-11/IGHV4-39/IGLV2-11/IGHV1-69/IGKV3-11/IGHV3-13/IGLV6-57/IGHV2-5/IGLV3-27/IGKV1D-39/IGHV3-48/IGKV1-17/IGHV4-59/IGKV1-33/IGLV1-47/IGLV2-14/FCGR3A/IGLV3-25/IGKV2-30/IGHV2-70/IGHV3-53/IGKV1D-16/IGKV1D-12/IGHV3-7/FCGR1A/IGKV3D-20/IGHV1-46/IGKV3-20/IGKV1D-33 |
| REACTOME_INITIAL_TRIGGERING_OF_COMPLEMENT | 79 | 1.75906808 | 0.02036847 | 0.01908628 | IGKV1-5/IGHV3-23/IGLV3-21/IGKV1-12/IGHV3-33/IGKV2D-40/IGKV4-1/IGHV3-30/IGHG1/IGHV1-2/IGHV4-34/IGKV2D-28/IGKV1-16/IGLV2-23/IGLV3-19/IGLV1-44/IGKV3-15/IGLV1-40/IGHV3-11/IGHV4-39/IGLV2-11/IGHV1-69/IGKV3-11/IGHV3-13/IGLV6-57/IGHV2-5/IGLV3-27/IGKV1D-39/MBL2/IGHV3-48/IGKV1-17/IGHV4-59/IGKV1-33/IGLV1-47/IGLV2-14/IGLV3-25/IGKV2-30/IGHV2-70/IGHV3-53/IGKV1D-16/IGKV1D-12/IGHV3-7/IGKV3D-20/IGHV1-46/IGKV3-20/IGKV1D-33/C1S/IGLC3/IGLV1-51 |
| REACTOME_CREATION_OF_C4_AND_C2_ACTIVATORS | 71 | 1.87082818 | 0.02036847 | 0.01908628 | IGKV1-5/IGHV3-23/IGLV3-21/IGKV1-12/IGHV3-33/IGKV2D-40/IGKV4-1/IGHV3-30/IGHG1/IGHV1-2/IGHV4-34/IGKV2D-28/IGKV1-16/IGLV2-23/IGLV3-19/IGLV1-44/IGKV3-15/IGLV1-40/IGHV3-11/IGHV4-39/IGLV2-11/IGHV1-69/IGKV3-11/IGHV3-13/IGLV6-57/IGHV2-5/IGLV3-27/IGKV1D-39/MBL2/IGHV3-48/IGKV1-17/IGHV4-59/IGKV1-33/IGLV1-47/IGLV2-14/IGLV3-25/IGKV2-30/IGHV2-70/IGHV3-53/IGKV1D-16/IGKV1D-12/IGHV3-7/IGKV3D-20/IGHV1-46/IGKV3-20/IGKV1D-33/C1S/IGLC3/IGLV1-51 |
| REACTOME_ROLE_OF_LAT2_NTAL_LAB_ON_CALCIUM_MOBILIZATION | 71 | 1.87820858 | 0.02036847 | 0.01908628 | IGKV1-5/IGHV3-23/IGLV3-21/IGKV1-12/IGHV3-33/IGKV2D-40/IGKV4-1/IGHV3-30/IGHV1-2/IGHV4-34/IGKV2D-28/IGKV1-16/IGLV2-23/IGLV3-19/IGLV1-44/IGKV3-15/IGLV1-40/IGHV3-11/IGHV4-39/IGLV2-11/IGHV1-69/IGKV3-11/IGHV3-13/IGLV6-57/IGHV2-5/IGLV3-27/IGKV1D-39/IGHV3-48/IGKV1-17/IGHV4-59/IGKV1-33/IGLV1-47/IGLV2-14/IGLV3-25/IGKV2-30/LYN/IGHV2-70/IGHV3-53/IGKV1D-16/IGKV1D-12/IGHV3-7/IGKV3D-20/IGHV1-46/IGKV3-20/IGKV1D-33/FCER1G/IGLC3/IGLV1-51 |
| REACTOME_FCGR_ACTIVATION | 69 | 1.94242798 | 0.02036847 | 0.01908628 | IGKV1-5/IGHV3-23/IGLV3-21/IGKV1-12/IGHV3-33/IGKV2D-40/IGKV4-1/IGHV3-30/IGHG1/IGHV1-2/IGHV4-34/IGKV2D-28/IGKV1-16/IGLV2-23/IGLV3-19/IGLV1-44/IGKV3-15/IGLV1-40/IGHV3-11/IGHV4-39/IGLV2-11/IGHV1-69/IGKV3-11/IGHV3-13/IGLV6-57/IGHV2-5/IGLV3-27/IGKV1D-39/IGHV3-48/IGKV1-17/IGHV4-59/IGKV1-33/IGLV1-47/IGLV2-14/FCGR3A/IGLV3-25/IGKV2-30/LYN/IGHV2-70/IGHV3-53/IGKV1D-16/IGKV1D-12/IGHV3-7/FCGR1A/IGKV3D-20/IGHV1-46/IGKV3-20/IGKV1D-33/HCK/IGLC3/IGLV1-51/YES1/IGHG4/FCGR2A/IGLV3-1 |
| PID_INTEGRIN1_PATHWAY | 66 | 1.80042979 | 0.02036847 | 0.01908628 | FGG/FGA/FGB/COL11A1/PLAU/FN1/SPP1/ITGA11/COL11A2/TGFBI/THBS2/COL5A1/LAMC2/FBN1/COL5A2/PLAUR/CSPG4/COL1A2/VCAM1/F13A1/COL3A1/COL1A1/ITGA5/COL6A3/COL4A4/COL6A2/COL6A1/NID1/LAMA4/ITGA4/COL4A1/ITGB1/ITGAV/COL7A1/TNC/COL4A3/LAMC1/ITGA1/LAMB1/THBS1 |
| REACTOME_COLLAGEN_DEGRADATION | 64 | 1.73872477 | 0.02036847 | 0.01908628 | MMP1/MMP13/MMP20/COL11A1/MMP8/MMP12/MMP10/COL10A1/MMP7/COL11A2/COL6A5/COL12A1/COL8A1/COL26A1/MMP11/COL5A1/MMP9/COL5A3/COL5A2/MMP14/COL9A1/CTSK/COL1A2/COL13A1/COL8A2/COL3A1/COL1A1/COL6A3/COL4A4/COL6A2/COL6A1/MMP3/COL4A1/COL19A1/CTSB/COL7A1/COL4A2/CTSL/MMP2/ADAM9/COL4A3/COL15A1/COL9A3/MMP15/ADAM17 |
| REACTOME_ASSEMBLY_OF_COLLAGEN_FIBRILS_AND_OTHER_MULTIMERIC_STRUCTURES | 61 | 1.78551877 | 0.02036847 | 0.01908628 | MMP13/MMP20/COL11A1/COL10A1/MMP7/COL11A2/COL6A5/COL12A1/COL8A1/TLL2/COL5A1/LAMC2/LOXL2/MMP9/LOX/COL5A3/COL5A2/COL9A1/COL1A2/COL8A2/BMP1/COL24A1/PXDN/COL3A1/COL1A1/CTSV/COL6A3/COL4A4/COL6A2/COL6A1/MMP3/COL4A1/TLL1/CTSS/CTSB/COL7A1/COL4A2/CTSL |
| REACTOME_CD22_MEDIATED_BCR_REGULATION | 61 | 1.97911944 | 0.02036847 | 0.01908628 | IGKV1-5/IGHV3-23/IGLV3-21/IGKV1-12/IGHV3-33/IGKV2D-40/IGKV4-1/IGHV3-30/IGHV1-2/IGHV4-34/IGKV2D-28/IGKV1-16/IGLV2-23/IGLV3-19/IGLV1-44/IGKV3-15/IGLV1-40/IGHV3-11/IGHV4-39/IGLV2-11/IGHV1-69/IGKV3-11/IGHV3-13/IGLV6-57/IGHV2-5/IGLV3-27/IGKV1D-39/IGHV3-48/IGKV1-17/IGHV4-59/IGKV1-33/IGLV1-47/IGLV2-14/IGLV3-25/IGKV2-30/LYN/IGHV2-70/IGHV3-53/IGKV1D-16/IGKV1D-12/IGHV3-7/IGKV3D-20/IGHM/IGHV1-46/IGKV3-20/IGKV1D-33/IGLC3/IGLV1-51 |
| PID_INTEGRIN3_PATHWAY | 43 | 1.90091313 | 0.02045717 | 0.01916939 | FGG/FGA/FGB/PLAU/FN1/SPP1/TGFBI/IBSP/L1CAM/FBN1/PLAUR/SDC1/COL1A2/COL1A1/THY1/COL4A4/LAMA4/PVR/EDIL3/COL4A1/ITGAV/ITGB3/TNC/PDGFRB/COL4A3/LAMC1/LAMB1/THBS1/SPHK1 |
| REACTOME_COMPLEMENT_CASCADE | 114 | 1.60761556 | 0.03605859 | 0.03378871 | CFHR4/IGKV1-5/IGHV3-23/C8B/IGLV3-21/IGKV1-12/IGHV3-33/IGKV2D-40/IGKV4-1/IGHV3-30/C9/IGHG1/IGHV1-2/IGHV4-34/IGKV2D-28/CFHR3/IGKV1-16/IGLV2-23/IGLV3-19/IGLV1-44/C8A/IGKV3-15/IGLV1-40/IGHV3-11/IGHV4-39/IGLV2-11/IGHV1-69/IGKV3-11/CPB2/IGHV3-13/IGLV6-57/IGHV2-5/IGLV3-27/IGKV1D-39/MBL2/IGHV3-48/IGKV1-17/IGHV4-59/IGKV1-33/IGLV1-47/IGLV2-14/CR1/IGLV3-25/IGKV2-30/C5AR1/IGHV2-70/IGHV3-53/IGKV1D-16/IGKV1D-12/IGHV3-7/CD55/IGKV3D-20/CFHR5/CPN2/IGHV1-46/IGKV3-20/CFH/IGKV1D-33/C3AR1/C1S/IGLC3/IGLV1-51 |
| REACTOME_GLUCURONIDATION | 25 | 1.79223502 | 0.04031 | 0.03777249 | UGT1A7/UGT1A6/UGT1A8/UGT1A10/UGT2B10/UGT1A3/UGT3A2/UGT1A1/UGT2B7/UGT1A4/UGT1A9/UGT2A3 |
